# Intrinsic Capacity Predictors of Dementia and Mortality in the Sydney Memory and Ageing Study

**DOI:** 10.1002/gps.70156

**Published:** 2025-10-14

**Authors:** Katya Numbers, Suraj Samtani, Katja Hanewald, Jared Cheung, Perminder S. Sachdev, Henry Brodaty

**Affiliations:** ^1^ Centre for Healthy Brain Ageing (CHeBA) Discipline of Psychiatry & Mental Health School of Clinical Medicine University of New South Wales Sydney Australia; ^2^ School of Risk & Actuarial Studies University of New South Wales Sydney Australia; ^3^ Centre for Population Ageing Research (CEPAR) University of New South Wales Sydney Australia

**Keywords:** cognitive ageing, frailty, intrinsic capacity, risk factors

## Abstract

**Background:**

Intrinsic Capacity (IC) is a developing concept focussed on promoting healthy ageing by maintaining functional abilities. It has been proposed as a better predictor of outcomes like dementia and mortality, compared to traditional frailty measures that focus on deficits. This study aims to replicate the five‐factor structure of IC among older adults in Australia and to evaluate its effectiveness in predicting long‐term health outcomes, in comparison with existing frailty measures.

**Methods:**

IC scores were computed for 400 older adults aged 70 to 90 participating in the Sydney Memory and Ageing Study (MAS). Structural equation modelling, including second‐order confirmatory analysis, was used to compute the five IC domains. Cox proportional hazard models were used to compare the predictive value of the second‐order IC factor with the Frailty Phenotype and Frailty Index for dementia over 10 years and mortality over 12 years.

**Results:**

Factor loadings for the IC structure yielded five subgroups (cognitive function, psychological health, locomotive ability, sensory function, vital function) with one global factor. The structure had good fit (SRMR = 0.054, GFI = 0.966). IC was associated with lower hazard (risk) of dementia (HR = 0.567, *p* < 0.001) over 10‐year and lower hazard of mortality (HR = 0.649, *p* < 0.001) over 12‐year, controlling for age, sex, and education. Finally, IC explained additional variance beyond the Frailty Phenotype when predicting both incident dementia and mortality risk. Compared with the Frailty Index, IC contributed additional variance for dementia risk but not for mortality.

**Conclusion:**

Evaluation of a person's IC at baseline explains additional variance compared to traditional frailty measures when predicting the risk of future negative health outcomes such as dementia incidence and mortality.

## Key Points

- The World Health Organisation proposed Intrinsic Capacity as a more holistic measure of capacity than purely disability or frailty scales.
- This study replicated the five factors of the Intrinsic Capacity measure in an Australian community sample aged 70–90 years who were dementia‐free at baseline.
- Intrinsic capacity proved to be superior to frailty measures in predicting risk of dementia over 10 years of follow up and mortality over 12 years of follow up.

## 1 Introduction

As average life expectancy increases and the global population ages, understanding age‐related functional decline and vulnerability to disease is becoming increasingly important [1]. This knowledge is crucial for developing effective interventions and healthcare policies to improve the quality of life for older adults. Disease, disability and decline have traditionally been conceptualised as a frailty syndrome, which captures and characterises the multisystem changes that occur with age [2]. The two most commonly used measures of frailty are the Frailty Phenotype (FP) [3] and the Frailty Index (FI) [4]. These tools help clinicians and researchers identify individuals at risk of adverse health outcomes, enabling more targeted and proactive care [5].

The FP classifies older people into three categories: ‘Frail’, ‘Prefrail’, or ‘Robust’. This classification is based on the presence or absence of five binary indicators: unintentional weight loss, exhaustion, weakness (measured by grip strength), slow walking speed, and low physical activity. These indicators theoretically capture the multidimensional nature of ageing, assessing decline or impairment across various physical and functional domains to provide a comprehensive overview of an individual's health status [3]. Conversely, the FI is predicated on the notion that health deficits accumulate with age. FI is calculated as a ratio where an aggregate of age‐related deficits is compared to the total number deficits assessed. The deficits are dichotomously scored (present vs. not) and capture factors such as stroke and cancer, grip strength and reduced peak expiratory flow, and impairments in activities of daily living [4].

Recently, the suitability of frailty as the primary measure of ‘health’ in older adults has been questioned, as ageing does not inherently result in disease and disability [6, 7]. Frailty as a syndrome assumes that individuals will inevitably develop physical and non‐physical deficits [2]; it fails to consider aspects of health that enable individuals to maintain homoeostasis and retain function [8]. As the medical understanding of ageing moves from absence of disease to retention of function, a construct that classifies people by their deficits may be less clinically relevant given not every deficiency warrants an intervention [9]. Finally, despite decades of research and many iterations of frailty indices, there is still no consensus on a gold standard measurement for frailty, evidenced by the heterogeneity between the FP and FI [10, 11, 12, 13].

In 2015, the World Health Organisation (WHO) introduced the concept of Intrinsic Capacity (IC) as part of its ‘Healthy Ageing’ framework [9], which promotes ongoing wellbeing and retention of functional ability in older adults. IC as a more holistic concept captures a range of both positive and negative physical and mental attributes individuals can draw on over a lifetime [14]. IC offers a novel way to conceptualise health in late life by including health‐related aptitudes that allow one to maintain wellbeing in older age [15].

Using exploratory factor analysis, Beard and colleagues [16] found that IC was comprised of five domains (cognitive function, psychological health, locomotive ability, sensory function, and vital function), which capture a range of physical and psychological processes associated with functional independence. IC theoretically captures health over the lifetime, rather than summing deficits at baseline. Data can be collected from patients throughout their lives, allowing for the monitoring of their health trajectories before deficit thresholds are met [17]. This can inform targeted early interventions for health and functional independence [18]. Another advantage of IC over frailty indices is the positive focus on capacity, which avoids negative stereotypes associating ageing with inevitable physical, cognitive and functional decline [19].

Although IC has been found to predict outcomes such as visual impairment, malnutrition, and depressive symptoms [20, 21, 22], there has been some heterogeneity across studies in the variables and the number of factors used to construct IC [23]. While some studies have created an IC score that captures all five of the original domains [24, 25], others have omitted the psychological [26] or sensory domains [27]. Additionally, the measures used for each domain differ significantly between studies. For example, some studies assessed the cognition domain using only a brief screening tool for dementia [25], while other studies used comprehensive neuropsychological testing to measure cognition [24, 28]. Recently, more studies have emerged validating the original five factors proposed by Beard and colleagues in 2019 in a number of international cohorts [29, 30, 31, 32]; however, to date, no study has examined this in an older Australian cohort. And while there has been a recent and rapid increase in studies examining the association between IC and mortality [33, 34, 35] and IC and functional decline [35, 36, 37, 38], to our knowledge, only one study has examined IC as a predictor for dementia risk [39].

The current study had three aims: (1) replicate the five factor IC model as proposed by Beard et al. in a large, well‐characterised sample of older Australians; (2) determine whether individual factors and/or an overall IC factor is associated with increased risk of incident dementia over 10‐year and death over 12 years; and (3) determine whether an overall IC factor is more predictive of dementia and/or mortality risk, than the FI or FP.

## 2 Methods and Materials

### 2.1 Participants

Participants were 1037 community dwelling older adults aged 70–90 who participated in the Sydney Memory and Ageing Study (MAS) [40]. MAS participants were recruited from two local government areas in Sydney's Eastern suburbs from 2005 to 2007. Baseline exclusion criteria were (a) insufficient fluency in English to complete a psychometric assessment; (b) a Mini‐Mental State Examination [41] (MMSE) score of ≤ 24 after adjusting for age, education, and non‐English speaking background, and (c) diagnoses of a major neurological illness such as dementia, motor neuron disease, or progressive malignancy. More details regarding baseline characteristics have been published previously [40]. Baseline cardiovascular disease and other systemic conditions were not exclusion criteria but were captured within the study measures (IC vital function domain and FI components).

**FIGURE 1:**
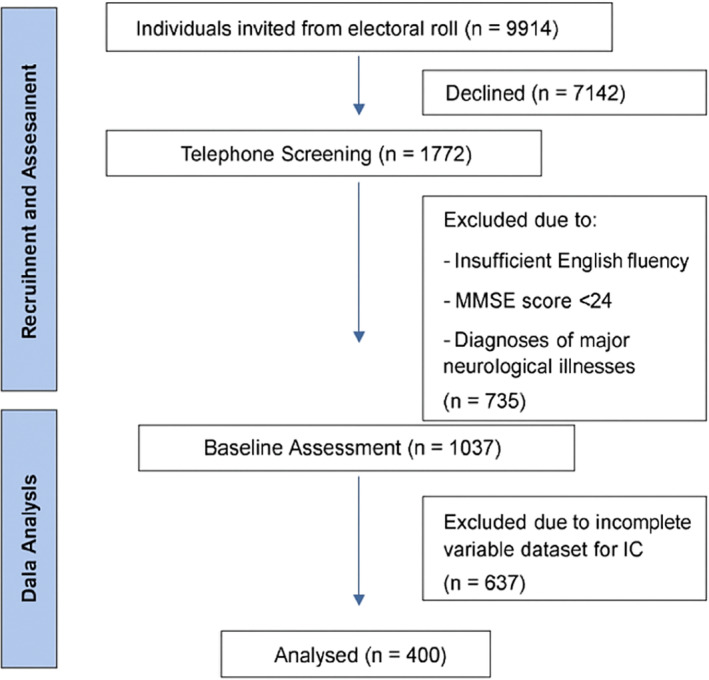
Participant flow^1^ diagram showing included and excluded participants in study.

Participants were assessed biennially for up to 12 years (called a ‘Wave’). At each Wave, participants completed questionnaires about their health, wellbeing, and mood, underwent a comprehensive neuropsychological assessment, and had a brief medical assessment. The basic demographics for the entire MAS baseline sample (*N* = 1037) are shown in Table S1. For the present study, participants were excluded from analyses if they did not have complete data for any of the variables included in the IC measure (*N* = 637), resulting in a final sample of 400. A consort diagram is presented in Figure 1.

#### 2.1.1 Frailty Phenotype (FP) Scores

FP scores were computed according to Fried et al.'s method using five criteria: (1) exhaustion was measured by the question ‘Do you feel full of energy?’ with ‘no’ responses receiving 1 point; (2) unintentional weight loss was approximated using BMI ≤ 18.5 as a proxy due to unavailable weight change data, receiving 1 point; (3) slowness was defined as performance in the bottom 20% for 6‐m walk time, receiving 1 point; (4) weakness was defined as performance in the bottom 20% for the timed sit‐to‐stand test, receiving 1 point; and (5) low physical activity was defined as the bottom 20% for weekly physical activity minutes (weighted by intensity), receiving 1 point. Total FP scores were calculated by summing individual criteria scores (range 0–5). Participants were then classified as Robust (score = 0), Prefrail (score = 1–2), or Frail (score ≥ 3).

#### 2.1.2 Frailty Index (FI) Scores

FI scores were calculated using the standard procedure outlined by Searle et al. [4]. The 66 individual selected variables from the MAS study were assigned cut‐off values according to the literature. If a participant met the threshold for a variable, they were assigned a score of 1. These dichotomous scores (0,1) were summed and divided by 66 to create a total FI ratio, with higher scores indicating more frailty. Supporting Information S1: Table S2 lists the definitions of all variables used to compute FI scores.

#### 2.1.3 Intrinsic Capacity (IC) Scores

IC scores were computed according to Beard et al. (2019)'s original methods [16]. We selected continuous or ordinal variables in our dataset that could potentially stratify individuals with high and low physical and cognitive ability. In addition, the variables chosen had to have some evidence of association with at least one of the original five domains of IC. The resultant list of variables included factors and/or equivalent used by Beard et al., as well as variables in the MAS dataset that fulfiled the previous two criteria. A comprehensive list of these variables appears in the Supporting Information S1.

### 2.2 Outcome Measures

Clinical diagnoses of dementia were available for Wave 1 to Wave 6 (10‐year follow‐up). At each wave, participants were brought to a consensus review meeting where a panel of neuropsychiatrists, psychogeriatricians, and neuropsychologists discussed all available clinical, neuropsychological, and imaging data to reach a consensus diagnosis. A diagnosis of dementia was based on the Diagnostic and Statistical Manual of Mental Disorders, Fourth Edition [42]: Participants with complete data who did not meet the criteria for a dementia were classified as ‘not dementia’.

Data on mortality were collected via three avenues: (1) interview/phone call to the relatives or nominated informant of the participants; (2) the Ryerson Index, an online repository for obituaries collected from newspapers across Australia; and (3) the Australian Institute of Health and Welfare's (AIHW) National Death Index (NDI) data repository. Data on mortality were available for Wave 1 to Wave 7 (12‐year follow‐up).

### 2.3 Statistical Analysis

A two‐tailed Pearson's correlation matrix was used to investigate the multicollinearity between all potential variables. Variables with *r* > 0.85 or extreme skewness > 3 were excluded prior to factor modelling. All IC, FP, and FI variables were computed using data from participants' baseline assessments (Wave 1) only. Follow‐up data from subsequent waves were used to ascertain time to incident dementia over 10‐year and time to death over 12‐year.

#### 2.3.1 Factor Analysis

A second order confirmatory factor analysis (CFA) tested the five‐factor IC structure (cognition, psychological health, locomotion, sensory function and vital function) and yielded a second‐order IC factor. Models were estimated using unweighted least‐squares (ULS). Because ULS does not provide root mean square error of approximation (RMSEA) or chi‐square statistics that are directly comparable under non‐normality, we report the standardised root mean square residual (SRMR) as the primary fit index and also provide the goodness‐of‐fit (GIF) and normed fit index (NFI) where available. The resulting IC factor score was transformed to a z‐score (higher = greater capacity). CFA required complete cases; therefore, IC scores were available for 400 participants with no missing data on IC indicators (observed range in current sample: −1.91 to 2.26).

#### 2.3.2 Survival Analyses

We fitted six Cox proportional hazards models to evaluate the predictive value of IC for incident dementia (over 10‐year) and all‐cause mortality (over 12‐year). For each outcome we estimated: (a) IC alone; (b) FP then IC; and (c) FI then IC. All models were adjusted for age, sex, and education. Step 1 included covariates; Step 2 added the frailty measure (FP as continuous 0–5 score, per 1‐point increase; FI as the count of deficits 0–66, per 1‐deficit increase); Step 3 added the global IC z‐score. Results are reported as hazard rations (HRs_ with 95% CIs.

Statistical significance was defined as *p* < 0.05. Statistical analyses were conducted using IBM SPSS Statistics 30 SAS 9.4. Descriptive statistics were performed to characterise cohort demographics.

### 2.4 Ethics

This study was approved by the University of New South Wales Human Research Ethics Committee (HC: 200671, 05037, 09382, 14327, 90626) in accordance with the National Statement on Ethical Conduct in Human Research (2007) and the Declaration of Helsinki. Informed written consent were obtained from all participants prior to participation.

## 3 Results

The sample for the current study comprised 400 MAS participants with complete data for all IC variables (see Table 1 for sample characteristics). They were aged 70–89 years, 46.8% were male, and their average education was 11.7 years. Participants' mean IC z‐score was 0.09 (SD = 0.67) and mean FI score was 20.30 (SD = 6.45). For the FP, 34.3% of participants were classified as Robust, 55.5% as Prefrail, and 7.0% as Frail. Compared to the excluded 637 participants, the current sample was younger (*p <* 0.001), but not significantly more likely to be female or to have less education *(p*'s > 0.05). Supporting Information S1: Table S1 provides a detailed comparison of included and excluded participants on baseline characteristics.

**TABLE 1:** Demographic characteristics of the MAS study sample at baseline (Wave 1) after exclusion (*N* = 400).

Characteristics	Mean or *N*	SD or %	Range
Age (years)	78.38	4.56	70–89
Sex (*N*, % male)	187	46.8%	—
Education (years)	11.72	3.55	4–24
Attention & processing speed	0.04	1.01	−4.03–3.65
Language	0.08	1.02	−3.35–2.53
Memory	0.09	1.01	−3.20–2.58
Executive function	0.06	0.96	−3.90–2.68
Visuospatial	0.15	0.99	−3–3
K10 total^a^	12.91	3.17	8–36
GAS total	1.07	1.82	0–8
GDS total	2.04	1.89	0–15
6m walk (sec)	8.65	2.35	4–30
Sit to stand test	16.07	4.70	7–43
Lateral stability	20.16	10.98	0–30
CVD risk	16.99	3.43	7–28
Forced expiratory volume	1.98	0.58	0.74–4.0
Grip strength	27.52	10.27	4–80
Subjective hearing	1.54	0.71	1–4
Subjective vision	3.91	0.30	1–4
Visual acuity	17.01	12.69	4.80–60.0
FI score	20.30	6.45	7–48
FP categories (*N*, %)^b^			
Robust (score = 0)	137	34.3%	—
Pre‐frail (score = 1–2)	222	55.5%	—
Frail (score ≥ 3)	28	7.0%	—
IC Score (second order, z)	0.09	0.67	−1.91–2.26

*Note:* K‐10 = Kessler Psychological Distress Scale – 10 item; GAS = Goldberg Anxiety Scale; GDS = Geriatric Depression Scale; 6 m walk (sec) = time taken to walk 6 m in seconds; CVD = cardiovascular disease; FI = Frailty Index; FP = Frailty Phenotype; IC = Intrinsic Capacity.

^a^ K‐10 scored 0–4 per item (total 0–40); values differ from the 10–50 convention due to recoding.

^b^ *N* = 387.

**FIGURE 2:**
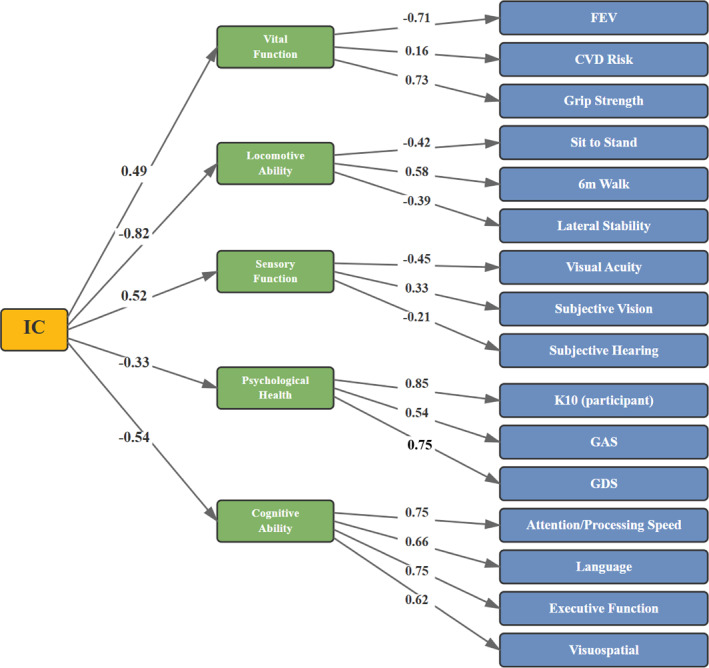
Second‐order structural model with factor loadings, estimated using unweighted least squares (ULS) factoring. Standardised loadings are presented for all observed variables onto their respective first‐order latent factors, and for first‐order factors onto the second‐order IC global construct.

For the first‐order CFA, estimated with ULS, fit was acceptable for the five‐favor model (SRMR = 0.075, GFI = 0.99, NFI = 0.98). Likewise, the second order CFA with the five factors loading onto a global IC factor also had a good fit (SRMR = 0.05, GFI = 0.96, NFI = 0.90). These five factors, which aligned with the Beard et al. model, were as follows: Cognitive function, psychological health, locomotive ability, sensory function, and vital function. Results from the second order CFA are presented in Figure 2.

Cox proportional hazards models adjusted for age, sex and education with dementia risk as the outcome variable and IC, FP and FI as the predictor variables are presented in Table 2. After controlling for relevant covariates, higher IC scores were associated with a lower risk of incident dementia (HR = 0.57; 95% CI: 0.40–0.80; *p* = 0.001; Table 2a). In stepwise models, IC contributed additional variance beyond FP (IC: HR = 0.48, 95% CI 0.32–0.71, *p* < 0.001; Table 2b) and beyond the FI (IC: HR = 0.52, 95% CI 0.35–0.77, *p* = 0.001; Table 2c). Participant age was positively correlated with dementia risk across models (i.e., even after adding IC in the final step). Male sex became significant in the FP and FI models when IC was added. Each one‐unit increase in IC corresponded to a 43% lower hazard of incident dementia.

**TABLE 2:** Cox proportional hazard models with IC, FP, and FI as the predictor variables and mortality as the outcome variable.

Model 1					Model 2				Model 3			
Variables	HR	95% CI		*p*	HR	95% CI		*p*	HR	95% CI		*p*
a. Intrinsic capacity												
Age	1.134	1.076	1.195	**<** **0.001**	1.095	1.034	1.16	**0.002**				
Sex	1.449	0.914	2.299	0.115	1.81	1.118	2.93	**0.016**				
Education	0.987	0.926	1.052	0.696	1.015	0.951	1.083	0.657				
Intrinsic capacity					0.567	0.400	0.803	**0.001**				
b. Frailty phenotype and intrinsic capacity												
Age	1.128	1.069	1.189	**<** **0.001**	1.13	1.069	1.193	**<** **0.001**	1.092	1.029	1.157	**0.003**
Sex	1.459	0.914	2.328	0.113	1.456	0.912	2.324	0.116	1.878	1.153	3.059	**0**.**011**
Education	0.991	0.93	1.057	0.790	0.99	0.929	1.056	0.769	1.019	0.955	1.087	0.568
Frailty phenotype					0.945	0.622	1.435	0.791	0.68	0.437	1.057	0.086
Intrinsic capacity									0.481	0.324	0.714	**<** **0.001**
c. Frailty index and intrinsic capacity												
Age	1.134	1.076	1.195	**<** **0.001**	1.126	1.064	1.192	**<** **0.001**	1.103	1.039	1.171	**0.001**
Sex	1.449	0.914	2.299	0.115	1.47	0.924	2.339	0.104	1.814	1.124	2.93	**0.015**
Education	0.987	0.926	1.052	0.696	0.991	0.929	1.058	0.788	1.013	0.949	1.081	0.698
Frailty index					1.013	0.972	1.056	0.541	0.979	0.935	1.025	0.362
Intrinsic capacity									0.519	0.349	0.77	**0.001**

*Note:* Bold values denote statistically significant.

Cox proportional hazards models with mortality risk at 12‐year follow‐up as the outcome variable and IC, FP and FI as the predictor variables are presented in Table 3. Higher IC was associated with lower hazard of all‐cause mortality (HR = 0.65, 95% CI 0.52–0.82, *p* < 0.001; Table 3a). When FP was entered, it predicted higher mortality (HR = 1.47, 95% CI 1.12–1.94, *p* = 0.006), but the FP effect attenuated and became non‐significant after adding IC (HR = 1.25, 95% CI 0.93–1.68, *p* = 0.143, while IC remained significant (HR = 0.71, 95% CI 0.55–0.91, *p* = 0.007; Table 3b). In models with FI, FI remained significant after adding IC (FI: HR = 1.05, 95% CI 1.02–1.08, *p* = 0.001; IC: HR = 0.79, 95% CI 0.61–1.02, *p* = 0.067), indicating no added variance from IC beyond FI for mortality (Table 3c). In sum, IC added variance beyond FP for dementia and mortality and beyond FI for dementia (but not mortality).

**TABLE 3:** Cox proportional hazards models with Intrinsic Capacity (IC), Frailty Phenotype (FP), and Frailty Index (FI) as predictor variables, and all‐cause mortality as the outcome.

Model 1					Model 2				Model 3			
Variables	HR	95% CI		*p*	HR	95% CI		*p*	HR	95% CI		*p*
a. Intrinsic capacity												
Age	1.137	1.098	1.177	**<** **0.001**	1.104	1.063	1.147	**<** **0.001**				
Sex	1.778	1.301	2.432	**<** **0.001**	2.068	1.498	2.855	**<** **0.001**				
Education	0.983	0.943	1.024	0.409	1.004	0.963	1.047	0.856				
Intrinsic capacity					0.649	0.517	0.815	**<** **0.001**				
b. Frailty phenotype and intrinsic capacity												
Age	1.134	1.095	1.174	**<** **0.001**	1.121	1.081	1.161	**<** **0.001**	1.102	1.061	1.144	**<** **0.001**
Sex	1.756	1.282	2.405	**<** **0.001**	1.782	1.302	2.439	**<** **0.001**	2.005	1.449	2.776	**<** **0.001**
Education	0.977	0.937	1.019	**0.286**	0.984	0.943	1.026	0.446	0.998	0.957	1.042	0.937
Frailty phenotype					1.474	1.119	1.942	**0.006**	1.248	0.928	1.679	0.143
Intrinsic capacity									0.707	0.55	0.909	**0.007**
c. Frailty index and intrinsic capacity												
Age	1.134	1.095	1.174	**<** **0.001**	1.104	1.065	1.145	**<** **0.001**	1.092	1.051	1.135	**<** **0.001**
Sex	1.756	1.282	2.405	**<** **0.001**	1.912	1.393	2.626	**<** **0.001**	2.068	1.49	2.869	**<** **0.001**
Education	0.977	0.937	1.019	0.286	0.998	0.957	1.04	0.910	1.006	0.964	1.05	0.781
Frailty index					1.063	1.037	1.09	**<** **0.001**	1.05	1.021	1.08	**0.001**
Intrinsic capacity									0.785	0.606	1.017	0.067

*Note:* Bold values denote statistically significant.

## 4 Discussion

This is the first study to replicate Beard's original 5‐factor model of IC in an older Australian cohort and builds on the growing body of literature regarding the validation and standardisation of IC. In addition to replicating Beard and colleagues' original IC factors [16], we found that an overall IC score was significantly associated with an increased risk of dementia over 10‐year and mortality over 12‐year. Furthermore, when entered together in regression models with FP and FI scores, the overall IC scores predicted additional unique variance in risk of dementia and mortality, over and above the FI and FP scores. Our findings that FP and FI predicted both outcomes are consistent with previous reports [43, 44, 45].

Although the scope of our study did not extend to examining individual IC domains' associations with dementia and mortality risk, our findings are consistent with other studies examining total IC scores as predictors of dementia and mortality [33, 34, 35, 36, 37, 38, 39, 40, 41, 42] and extend these findings over a much longer follow‐up period. Together, this provides good evidence that IC scores can accurately capture current, and predict long‐term, cognitive and physical health trajectories in older adults. Further, we demonstrated that IC had a stronger predictive ability for the risk of dementia than standard frailty measures.

The extra variance accounted by IC over the typical, deficit‐focused, frailty measures supports the notion that the maintenance of healthy ageing is a holistic concept that goes beyond physical deficits. The associations between mortality and physical performance on measures like mobility [46] and grip strength [47] as well as between dementia and measures of locomotion [48] and cardiovascular risk [49], have been well established and used in clinical assessments.

Our findings suggest that two standardized frailty measures—the FI and FP—provide complementary information about older adults' health trajectories. The FI, which quantifies accumulated deficits, serves as a sensitive measure of overall health burden and may be particularly effective for identifying short‐term mortality risk due to its alignment with end‐of‐life health decline. In contrast, IC assesses functional reserve across multiple domains and may better reflect an individual's capacity for maintaining health and responding to interventions over time. This complementary relationship suggests that while FI may indicate immediate risk, IC provides a broader assessment of potential for healthy ageing and successful intervention responses. The clinical value lies not in choosing between these approaches, but in understanding how they can inform different aspects of care planning for older adults.

Although a lower IC score was strongly associated with a higher risk of death, it did not account for additional variance in mortality risk beyond that explained by FI, which may be capturing more end‐of‐life variables compared to IC. The FI primarily assesses age‐associated deficits, which may more accurately predict mortality than the IC, which measures baseline health levels. A systematic review of 2617 studies found that the association between frailty and mortality became less prominent in studies with longer follow‐up periods [45]. This suggests that the FI's closer alignment with immediate health decline makes it a stronger predictor of short‐term mortality compared to the IC.

Key strengths of our study were comprehensive neuropsychological testing, expert clinical consensus diagnoses, and long follow‐up period. Participants underwent detailed physical, psychological, cognitive, and mental health assessments, as well as blood tests and informant interviews, which provided a unique capacity to test more variables within the IC construct. It also enabled us to explore the predictive value of IC for longer‐term outcomes such as dementia risk and mortality compared to other studies. The use of Cox proportional hazard models accounts for non‐random attrition in our sample and allowed for greater predictive power.

There are limitations to our research. Our study relied on data from the MAS study, which recruited using an opt‐in system from electoral rolls in a relatively more affluent and culturally homogenous area in south‐east Sydney. MAS participants were well‐educated, had a high average household income, and the overwhelming majority (93%) identified as White Australian/New Zealander or White Western‐European. The resultant cohort is therefore not fully representative of the wider older Australian population. Furthermore, the exclusion criteria of participants with dementia at baseline, which is when variables for the IC analyses were collected, could have contributed to the non‐normal distribution of several variables in our dataset, such as mood, physical activity and MMSE scores. We also used BMI ≤ 18.5 as a proxy for unintentional weight loss, which may not accurately capture recent weight changes as intended in the original FP definition. This measurement limitation may have led to misclassification of some participants. Future studies should utilize longitudinal weight data when available to better reflect the original Fried criterion.

A primary limitation is the potential for selection bias resulting from excluding a substantial portion of the original cohort (*n* = 637) due to missing data required for IC calculations. This exclusion is unavoidable with CFA methods but may have resulted in a non‐random subset of participants. For example, participants with higher levels of frailty or cognitive impairment higher levels of frailty or cognitive impairment were less likely to complete all assessments (see Supporting Information S1: Table S1). Future research could examine the feasibility of constructing and validating a shorter version of indicators that represent IC, making the formation of a composite score more accessible for clinicians.

## 5 Conclusion

In conclusion, our findings suggest IC offers a fundamentally different perspective from traditional frailty assessments. While frailty measures focus on deficit accumulation, IC captures functional reserve and recognizes that healthy ageing encompasses both the absence of deficits and the presence of functional abilities. IC's integration of multiple health domains—cognitive, psychological, locomotive, sensory, and vital function—makes it particularly valuable for person‐centred interventions aimed at maintaining and enhancing abilities rather than merely addressing deficits.

Further, our study provides strong evidence that IC serves as a comprehensive marker for healthy ageing, demonstrating unique predictive value for both dementia and mortality over 10 and 12 years of follow‐up, respectively. This predictive ability, extending beyond what traditional frailty measures can provide, positions IC as a valuable tool for early identification of individuals who may benefit from targeted interventions to maintain functional independence and prevent adverse outcomes. The WHO's Healthy Ageing framework, with IC at its core, therefore, represents a paradigmatic shift towards capacity‐focused rather than deficit‐focused health assessment in older adults.

## Author Contributions

Jared Cheung wrote the original draft, analysed and interpreted the data, and reviewed the literature as part of his Honours project. Henry Brodaty conceived and supervised the study. Perminder S. Sachdev oversaw all clinical consensus meetings and dementia diagnoses. Katya Numbers and Suraj Samtani co‐supervised the study and provided statistical assistance and feedback on all drafts of the original thesis. Katya Numbers, Suraj Samtani, Katja Hanewald and Henry Brodaty wrote and edited the current draft of this manuscript and completed additional statistical analysis.

## Ethics Statement

The Memory and Ageing Study (MAS) was approved by the University of New South Wales Human Research Ethics Committee (HC: 200671, 05037, 09382, 14327, 90626) in accordance with the National Statement on Ethical Conduct in Human Research (2007) and the Declaration of Helsinki. Informed written consent were obtained from all participants prior to participation. The current study analyses were approved by the same committee.

## Consent

The authors have nothing to report.

## Conflicts of Interest

Henry Brodaty is or has been an advisory board member or consultant to Biogen, Eisai, Eli Lilly, Medicines Australia, NovoNordisk, Roche and Skin2Neuron. Perminder S. Sachdev has been on the expert advisory panels for Biogen, Eli Lilly, NovoNordisk and Roche Australia.

## Supporting information


Supporting Information S1

## Data Availability

The terms of consent for research participation stipulate that an individual's data can only be shared outside of the MAS investigators group if the group has reviewed and approved the proposed secondary use of the data. This consent applies regardless of whether data have been de‐identified. Access is mediated via a standardised request process managed by the CHeBA Research Bank, who can be contacted at ChebaData@unsw.edu.au.

## Endnotes

^1^
In Cox proportional hazards models, FP entered as a continuous score (0–5; per 1‐point increase). FI entered as count of deficits (0–66; per 1‐deficit increase). Table 1 presents FP as categories (Robust/Prefrail/Frail) for descriptive clarity; FI descriptives are shown as a proportion (deficits/66).
