# Supplementary material for: Intrinsic Capacity Predictors of Dementia and Mortality in the Sydney Memory and Ageing Study
Source: Int J Geriatr Psychiatry. 2025 Oct 14;40(10):e70156. doi: 10.1002/gps.70156 (PMC12560156; doi:10.1002/gps.70156)
Supplement: Supplementary file 1 — Supporting Information S1 [file GPS-40-0-s001.docx]

**Supplementary – Tables (S1 – S4) and Text (ST)**

| *Supplementary Table 1 (S1): Demographic characteristics of MAS sample at baseline (N=1037)* | | | | | |
| --- | --- | --- | --- | --- | --- |
| **Characteristics** | **N (Missing)** | **Mean** | **SD** | **Range** | |
| **Age (years)** | 1037 (0) | 78.33 | 4.81 | 70 – 90 | |
| **Gender (% male)** | 1037 (0) | n/a | .50 | n/a | |
| **Education (years)** | 1037 (0) | 11.60 | 3.47 | 3 – 24 | |
| ***Baseline IC Variables*** | | | | | |
| **Attention & Processing Speed** | 950 (83) | -.06 | 1.03 | -4.27 – 2.68 | |
| **Language** | 1030 (7) | -.18 | 1.12 | -4.9 – 3.11 | |
| **Memory** | 1021 (16) | -.13 | 1.08 | -5.3 – 3.66 | |
| **Executive Function** | 1026 (11) | -.11 | 1.04 | -3.20 –2.73 | |
| **Visuospatial** | 1034 (3) | -.07 | 1.02 | -3 – 3 | |
| **K10 (participant)** | 1022 (15) | 13.36 | 3.65 | 8 – 37 | |
| **Goldberg Anxiety Scale** | 1007 (30) | 1.12 | 1.87 | 0 – 8 | |
| **Geriatric Depression Scale** | 1032 (5) | 2.29 | 2.08 | 0 – 15 | |
| **6m walk (sec)** | 1007 (30) | 9.23 | 2.83 | 4 – 30 | |
| **Sit to Stand test** | 925 (112) | 16.81 | 5.64 | 7 – 65 | |
| **Lateral stability** | 1020 (17) | 18.90 | 11.67 | 0 – 30 | |
| **CVD risk** | 998 (39) | 17.31 | 3.52 | 7 – 28 | |
| **Forced Expiratory Volume** | 530 (507) | 1.97 | .56 | .65 – 4.0 | |
| **Grip Strength** | 595 (442) | 27.43 | 11.08 | 4 – 90 | |
| **Subjective Hearing** | 1036 (1) | 1.54 | .72 | 1 – 4 | |
| **Subjective Vision** | 1032 (5) | 3.88 | .39 | 1 – 4 | |
| **Visual Acuity** | 1001 (36) | 18.43 | 13.62 | 3.80 – 60.0 | |
| ***Baseline Predictor Variables*** | | | | | |
| **Frailty Phenotype** | 928 (109) | 1.80 | .63 | 1 | – 3 |
| **Frailty Index** | 1037 (0) | 20.93 | 6.76 | 7 | – 48 |

| *Supplementary Table 2 (S2): Characteristics of participants included and excluded from the analysis at baseline.* | | | |
| --- | --- | --- | --- |
| **Variable** | **Included (n=400)** | **Excluded (n=637)** | |
|  | **Mean (SD) or N (%)** | **Mean (SD) or N (%)** | **p-value** |
| **Demographics** | | | |
| Age | 77.38 (4.56) | 78.93 (4.87) | <0.001 |
| Sex (% male) | 53.0% | 56.0% | 0.329 |
| Education (years) | 11.71 (3.55) | 11.53 (3.43) | 0.401 |
| **Cognitive Factors** | | | |
| Attention | 0.04 (1.01) | -0.24 (1.12) | <0.001 |
| Language | 0.08 (1.02) | -0.34 (1.15) | <0.001 |
| Executive Function | 0.06 (0.96) | -0.15 (1.07) | 0.002 |
| Visuospatial | 0.15 (0.99) | -0.21 (1.02) | <0.001 |
| Memory | 0.09 (1.01) | -0.24 (1.04) | <0.001 |
| **Psychological Factors** | | | |
| GAS Total | 1.07 (1.82) | 1.15 (1.91) | 0.522 |
| GDS Total | 2.04 (1.89) | 2.44 (2.19) | 0.003 |
| K10 Total | 12.91 (3.18) | 13.64 (3.89) | 0.002 |
| **Locomotion Factors** | | | |
| 6-minute walk | 8.64 (2.35) | 9.62 (3.05) | <0.001 |
| Sit-to-Stand | 16.07 (4.70) | 17.37 (6.20) | <0.001 |
| Lateral Stability | 20.16 (10.98) | 18.09 (12.03) | 0.006 |
| **Vitality Factors** | | | |
| Grip Strength | 27.52 (10.27) | 27.24 (12.62) | <0.001* |
| FEV | 1.98 (0.58) | 1.93 (0.52) | 0.339 |
| CVD Risk | 16.99 (3.43) | 17.52 (3.56) | 0.020 |
| **Sensory Factors** |  |  |  |
| Visual Acuity | 17.01 (12.69) | 19.37 (14.14) | 0.007 |
| Subjective Hearing | 1.53 (0.71) | 1.54 (0.73) | 0.952 |
| Subjective Vision | 1.09 (0.30) | 1.13 (0.43) | 0.148 |
| Frailty Phenotype | 1.72 (0.59) | 1.87 (0.64) | <0.001 |
| Frailty Index | 0.19 (0.06) | 0.20 (0.06) | 0.315 |
| *Note:* * p < 0.05 indicates significant difference between groups | | | |

| *Supplementary Table 3a (S3a.) Cox proportional hazards models examining the Frailty Phenotype (FP) as a categorical predictor of dementia risk.* | | | | | | | | | | | | |
| --- | --- | --- | --- | --- | --- | --- | --- | --- | --- | --- | --- | --- |
| **Variable** | **Model 2 - Original (Continuous FP)** | | | **Model 2 - Sensitivity (Categorical FP)** | | | **Model 3 - Original (Continuous FP + IC)** | | | **Model 3 - Sensitivity (Categorical FP + IC)** | | |
|  | **HR** | **95% CI** | ***p*** | **HR** | **95% CI** | ***p*** | **HR** | **95% CI** | ***p*** | **HR** | **95% CI** | ***p*** |
| Age | 1.130 | 1.069–1.193 | <.001 | 1.122 | 1.082–1.163 | <.001 | 1.092 | 1.029–1.157 | .003 | 1.105 | 1.064–1.148 | <.001 |
| Sex (Male) | 1.456 | 0.912–2.324 | .116 | 0.559 | 0.408–0.764 | <.001 | 1.878 | 1.153–3.059 | .011 | 0.515 | 0.373–0.710 | <.001 |
| Education | 0.990 | 0.929–1.056 | .769 | 0.984 | 0.943–1.026 | .438 | 1.019 | 0.955–1.087 | .568 | 0.997 | 0.956–1.041 | .907 |
| Frailty Phenotype (Continuous) | 0.945 | 0.622–1.435 | .791 | – | – | – | 0.680 | 0.437–1.057 | .086 | – | – | – |
| Prefrail vs Robust | – | – | – | 1.356 | 0.945–1.945 | .098 | – | – | – | 1.250 | 0.865–1.807 | .235 |
| Frail vs Robust | – | – | – | 2.344 | 1.332–4.123 | .003 | – | – | – | 1.730 | 0.927–3.227 | .085 |
| Intrinsic Capacity | – | – | – | – | – | – | 0.481 | 0.324–0.714 | <.001 | 1.399 | 1.047–1.869 | .023 |
| *Notes:* FP was entered as a three-level categorical variable (Robust = reference, Prefrail, Frail). Models adjusted for age, sex, and education. Results are presented as hazard ratios (HR) with 95% confidence intervals (CI). Continuous FP scored 0-5; Categorical FP: Robust (≤1), Prefrail (2), Frail (≥3) | | | | | | | | | | | | |

| *Supplementary Table 3b (S3b) Cox proportional hazards models examining the Frailty Phenotype (FP) as a categorical predictor of mortality risk.* | | | | | | | | | | | | |
| --- | --- | --- | --- | --- | --- | --- | --- | --- | --- | --- | --- | --- |
| **Variable** | **Model 2 - Original (Continuous FP)** | | | **Model 2 - Sensitivity (Categorical FP)** | | | **Model 3 - Original (Continuous FP + IC)** | | | **Model 3 - Sensitivity (Categorical FP + IC)** | | |
|  | **HR** | **95% CI** | **p** | **HR** | **95% CI** | **p** | **HR** | **95% CI** | **p** | **HR** | **95% CI** | **p** |
| **Age** | 1.13 | 1.069-1.193 | <.001 | 1.122 | 1.082-1.163 | <.001 | 1.092 | 1.029-1.157 | .003 | 1.105 | 1.064-1.148 | <.001 |
| **Sex (Male)** | 1.456 | 0.912-2.324 | .116 | 0.559 | 0.408-0.764 | <.001 | 1.878 | 1.153-3.059 | .011 | 0.515 | 0.373-0.710 | <.001 |
| **Education** | 0.99 | 0.929-1.056 | .769 | 0.984 | 0.943-1.026 | .438 | 1.019 | 0.955-1.087 | .568 | 0.997 | 0.956-1.041 | .907 |
| **Frailty Phenotype (Continuous)** | 0.945 | 0.622-1.435 | .791 | - | - | - | 0.68 | 0.437-1.057 | .086 | - | - | - |
| **Prefrail vs Robust** | - | - | - | 1.356 | 0.945-1.945 | .098 | - | - | - | 1.250 | 0.865-1.807 | .235 |
| **Frail vs Robust** | - | - | - | 2.344 | 1.332-4.123 | .003 | - | - | - | 1.730 | 0.927-3.227 | .085 |
| **Intrinsic Capacity** | - | - | - | - | - | - | 0.481 | 0.324-0.714 | <.001 | 1.399 | 1.047-1.869 | .023 |
| *Notes:* FP was entered as a three-level categorical variable (Robust = reference, Prefrail, Frail). Models adjusted for age, sex, and education. Results are presented as hazard ratios (HR) with 95% confidence intervals (CI). Continuous FP scored 0-5; Categorical FP: Robust (≤1), Prefrail (2), Frail (≥3) | | | | | | | | | | | | |

## *Supplementary Text (ST): Variables Included in Intrinsic Capacity Global Score*

We considered variables that had also been identified by Beard et al.24, as well as other variables that might potentially give an objective estimate of one of the IC domains.

Variables that have been used by the ELSA study and/or equivalents are shown below:

- Attention and Processing Speed: This was a z-score compiled from results scored from the Trail Making Test A and Digital Substitution Test.

- Language: This was a z-score compiled from results scored from the 60-item Boston Naming test and Animal Naming Tests, which measure confrontational word retrieval and semantic fluency
- Memory: This was a z-score compiled from results scored from the Rey Auditory Verbal Learning Tests, Logical Memory Delay Test, and Benton Visual Retention Test, which test for a wide variety of higher order cognitive function
- Geriatric Depression Scale: 15-item self-report measure of depression in older adults
- 6 Metre walk: Participants were measured on the time taken to walk 6 metres in a straight line. Walking aids were permitted
- Sit to Stand test: participants were asked to sit in a chair and were timed on the amount of taken to stand up and sit down five times in a row.
- Forced Expiratory Volume: Participants were measured 3 times using a peak flow meter, with the maximum value recoded for each participant used in the analysis.
- Grip Strength: Handgrip strength was measured 3 times on each hand with a dynamometer, with measurements starting on the dominant hand and alternating between dominant and non-dominant. The maximum value recorded for each participant was used in the analysis.
- Subjective Hearing: Participants were asked whether their hearing (with hearing aids if applicable) was adequate for everyday purposes
- Subjective Vision: Participants were asked whether their hearing (with hearing aids if applicable) was adequate for everyday purposes

Our study included the following new variables:

- Executive Function: This was a z-score compiled from results scored from Trail Making Test B and FAS tests, neuropsychological screens for visual attention and task switching, as well as phonemic verbal fluency
- Visuospatial: This was a z-score compiled from results scored from the block design test, a test designed to measure spatial visualisation
- Kessler Psychological Distress Scale 10 (K10): A short questionnaire for both the patient and carer that was designed to be a screening measure of anxiety and mood disorders.
- Goldberg Anxiety Scale: An 18-item self-report symptom inventory designed as a simple diagnostic tool for anxiety
- CVD risk: an 8-item risk appraisal tool that was computed based on the Framingham Stroke Study. Participants were scored based on their number of cardiovascular risk factors.
- Visual Acuity: Participants were assessed using a handheld Snellen Chart
